# Reduction in neural activation to high-calorie food cues in obese endometrial cancer survivors after a behavioral lifestyle intervention: a pilot study

**DOI:** 10.1186/1471-2202-13-74

**Published:** 2012-06-25

**Authors:** Nora L Nock, Anastasia Dimitropolous, Jean Tkach, Heidi Frasure, Vivan vonGruenigen

**Affiliations:** 1 Department of Epidemiology and Biostatistics, Case Western Reserve University, Wolstein Research Building, 2103 Cornell Road, Cleveland, OH, 44106-7281, USA; 2 Case Comprehensive Cancer Center, Case Western Reserve University, Cleveland, OH, 44106, USA; 3 Department of Psychological Sciences, Case Western Reserve University, Cleveland, OH, 44106, USA; 4 Case Center for Imaging Research, Department of Radiology, Case Western Reserve University, Cleveland, OH, 44106, USA; 5 University Hospitals Case Medical Center, Cleveland, OH, 44106, USA; 6 Department of Reproductive Biology, University Hospitals Case Medical Center, Case Western Reserve University, Cleveland, OH, 44106, USA

**Keywords:** Obesity, Endometrial cancer, fMRI, Reward, High-calorie foods, Lifestyle intervention

## Abstract

**Background:**

Obesity increases the risk of endometrial cancer (EC) and obese EC patients have the highest risk of death among all obesity-associated cancers. However, only two lifestyle interventions targeting this high-risk population have been conducted. In one trial, food disinhibition, as determined by the Three-Factor Eating Questionnaire, decreased post-intervention compared to baseline, suggesting an increase in emotional eating and, potentially, an increase in food related reward. Therefore, we evaluated appetitive behavior using functional magnetic resonance imaging (fMRI) and a visual food task in 8 obese, Stage I/II EC patients before and after a lifestyle intervention (Survivors in Uterine Cancer Empowered by Exercise and a Healthy Diet, SUCCEED), which aimed to improve nutritional and exercise behaviors over 16 group sessions in 6 months using social cognitive theory.

**Results:**

Congruent to findings in the general obese population, we found that obese EC patients, at baseline, had increased activation in response to high- vs. low-calorie food cues after eating a meal in brain regions associated with food reward (insula, cingulate gyrus; precentral gyrus; whole brain cluster corrected, p < 0.05). At 6 months post-intervention compared to baseline, we observed decreased activation for the high-calorie vs. non-food contrast, post-meal, in regions involved in food reward and motivation (posterior cingulate, cingulate gyrus, lateral globus pallidus, thalamus; claustrum; whole brain cluster corrected, p < 0.05).

**Conclusions:**

Our preliminary results suggest behavioral lifestyle interventions may help to reduce high-calorie food reward in obese EC survivors who are at a high-risk of death. To our knowledge, this is the first study to demonstrate such changes.

## Background

Endometrial cancer (EC) is the fourth leading cancer in women in the U.S. with approximately 43,470 new cases diagnosed annually [1]. Obesity, which has been rising in epidemic proportions over the last several decades in the U.S., is one of the strongest risk factors for EC [2,3], particularly Type I (estrogen-dependent) forms [4,5]. Interestingly, there is evidence that rates of Type I EC have been increasing [6]. Obese (body mass index (BMI) ≥ 30.0 kg/m^2^) women have a 1.7 to 4.5 fold greater risk of developing EC than normal weight women (BMI 25- < 30.0 kg/m^2^) [7-12]. Adult weight gain has also been dose-dependently associated with EC risk, whereby a 1.8 fold increased EC risk was observed among women whose BMI increased 5 to 15% and a 4.1 fold increased EC risk was found for women whose BMI increased 35% or more [9].

The majority of EC patients are overweight or obese with obesity rates estimated between 38%-65% [13,14], which, even on the lower end of the range, appears to be slightly higher than the estimated rates of obesity in the general adult female population in the U.S. at 35.5% [15]. Furthermore, EC patients have the highest risk of death among all of the obesity-associated cancers with a 2.5 fold increase in the risk of death for Class I obesity (BMI: ≥ 30.0- < 35.0 kg/m^2^) up to over a 6-fold increase in the risk of death for Class III obesity (BMI: ≥ 40.0 kg/m^2^) [16]. Von Gruenigen et al. [14] also observed decreased survival in EC patients with Class III obesity and, found that 67% of EC deaths were attributed to non-cancer related causes [14]. Thus, EC survivors appear to be dying from the co-morbidities associated their obesity (e.g., diabetes, cardiovascular and pulmonary diseases) not from their cancer [14,17]. Moreover, EC survivors do not appear to make spontaneously lifestyle changes during the ‘teachable moment’ of a cancer diagnosis [18] like other types of cancer survivors (e.g., post-menopausal breast cancer survivors) [19], which emphasizes the necessity of conducting lifestyle interventions in EC survivors.

The exact mechanisms driving the association between obesity and EC are not known but several biologically plausible hormonal, metabolic and inflammatory related mechanisms have been proposed. Perhaps, the most prevailing, is the theory that obesity, through the upregulation of aromatase in peripheral adipocytes, induces higher estrogen levels, which are unopposed by progesterone and bind to the estrogen receptor, leading to dysregulated endometrial cell proliferation and malignant transformation [3,4,20]. This may be further exacerbated in obese women because they have lower levels of sex-hormone binding globulin (SHBG) [21], which would not be available to bind to and render the estrogen biologically inactive. In addition to effects on uterine tissue, estradial affects a diverse array of brain functions and plays a key role in regulating gonadotropin-releasing hormone (GnRH) neurons and, could be involved with excitatory and inhibitory neuronal function in the hypothalamus [22,23]. Other putative mechanisms for the association between obesity and endometrial cancer involve insulin, which is key in regulating glucose metabolism, and insulin-like growth factor (IGF) signaling, which, when bound to its receptor, can lead to aberrant cancer cell proliferation. Inflammatory factors, such as the pro-inflammatory cytokine, TNF-α, can disrupt insulin signaling and, therefore, also lead to aberrant cell growth [3,4]. Leptin, which is secreted by adipocytes in proportion to the amount of fat mass stored, is most commonly known for its role in the regulation of energy intake and energy expenditure [24]; however, leptin may also act as a growth factor and has been shown to stimulate human endometrial cancer cell proliferation [25]. It is noteworthy that significantly higher levels of leptin have been found in obese endometrial cancer patients compared to those with a normal endometrium [26].

Even though obesity is one of the strongest risk factors for EC and obese EC patients have the highest risk of death among all cancer survivors, only two behavioral lifestyle interventions targeting EC patients have been reported [27,28]. In the first study, von Gruenigen and colleagues found that a six month, group behavioral lifestyle intervention involving 45 EC patients (23 randomized to treatment; 22 randomized to control) was successful in reducing EC survivor weight (3.5 kg, on average, in the treatment group) [27]. Using the Three-Factor Eating Questionnaire (TFEQ) [29], Von Gruenigen et al. [30] reported that EC patients who lost weight had improved eating restraint; however, “weight losers had a lower disinhibition score indicating an increased likelihood to overeat in the presence of disinhibitors”, which (assuming the items were not reversed scored) suggests an increase in emotional eating and, possibly an increase in non-homeostatic food drive and reward. In another behavioral intervention study, which included 33 gynecologic cancer patients (11 EC: 6 randomized to treatment; 5 randomized to control), Donnelly et al. [28] found a significant decrease in fatigue (the primary endpoint) at six months but they did not report on changes in weight loss or eating behavior.

Although questionnaires based on self-report are commonly used to evaluate changes in eating behavior in lifestyle interventions, these methods suffer from potential biases. The TFEQ is one of the most widely used measures of eating behavior but concerns have been raised about the TFEQ factor structure and validity [31-33]. To minimize bias from these issues, studies have used neuroimaging techniques to objectively evaluate appetitive behavior by measuring differential activation in response to visual food cues in brain regions that process and reinforce the rewarding value of food including the corticolimbic, hypothalamic, and midbrain circuits [34,35].

In obese individuals, high-calorie, palatable food stimuli may possess greater potency for activating the reward system and may trigger excessive motivation to non-homeostatic eating [36]. Higher BMI levels have been shown to positively correlate with higher high-calorie food cravings [37]. Obese women shown pictures of high-calorie foods were found to have increased activation in the dorsal striatum [38,39], a brain region implicated in reward anticipation and cue-induced drug craving and drug seeking [40]. Increased activation in obese compared to normal weight women has also been observed in regions associated with taste information processing (anterior insula, lateral orbitofrontal cortex), motivation (orbitofrontal cortex, OFC) and memory (posterior cingulate) [38,41]. We recently found that obese compared to normal weight adults had increased activation in frontal, temporal and limbic regions in response to high- and low-calorie food cues and greater activation in corticolimbic regions (lateral OFC, caudate, anterior cingulate) in response to high-calorie food cues after eating a standardized (750 kcal) meal, suggesting obese adults have sustained response to high-calorie food reward even after eating [42]. In addition, Martin et al. [43] found that obese compared to normal weight adults had increased activation in the medial prefrontal cortex after consuming a meal.

There are only a few studies that have utilized neuroimaging to objectively evaluate effectiveness of weight loss and to determine whether or not lifestyle interventions in obese individuals can successfully modify the obese patient’s response to food. Using positron emission tomography (PET), Le et al. [44] found that formerly obese women who successfully achieved weight loss (reduced BMI from ≥35 to ≤25 kg/m^2^), had greater activation in the dorsolateral prefrontal cortex (DLPFC) in response to a meal. Using functional magnetic resonance imaging (fMRI), McCaffery et al. [45] observed greater activation in successful weight losers (maintained ≥30 lb weight loss for ≥3 years) in response to high-calorie food vs. non-food pictures in the left superior frontal region and right middle temporal region, suggesting greater inhibitory control and greater visual attention to the high-calorie food cues. Phelan et al. [46] subsequently showed that weight loss maintainers (weight loss of ≥30 lbs for ≥3 years) differed with respect to their cognitive responses using a Food Stroop paradigm whereby a significantly slower reaction time was observed when naming high-calorie but not low-calorie foods and, they suggested future research should prospectively determine whether interventions can change such cognitive processes to better facilitate long-term weight control. We are only aware of one recently published study that prospectively examined neural response to food images before and after a behavioral lifestyle intervention in obese individuals; and, in this study, Murdaugh et al. [47] found that obese patients undergoing a 12-week behavioral intervention had decreased activation to high-calorie food pictures post- compared to pre-treatment in regions implicated in reward and attentional processes (medial prefrontal cortex, posterior cingulate, inferior parietal lobule, precuneus).

No prior studies have examined brain response to visual food cues in obese EC survivors, who appear to have higher rates of obesity than the general adult female population in the U.S. [13-15] and who are at a high risk of death due, predominantly to co-morbidities associated with their obesity [14,17]. Thus, we aimed to examine neural response to visual food cues under fasted and fed states using fMRI in obese EC patients before and after a lifestyle intervention (Survivors in Uterine Cancer Empowered by Exercise and a Healthy Diet, SUCCEED), which aimed to improve nutritional and exercise behaviors over 16 group sessions in 6 months (ClinicalTrials.gov Identifier: NCT00732173). We hypothesized that the neural response to high-calorie food cues in obese EC survivors would be similar to that found in the general obese population and that this response would be attenuated in the treatment group after receiving the intervention.

## Methods

### Study Participants

The study population consisted of 8 obese, Stage I/II EC patients enrolled in the treatment group of Wave 3 of the ‘Survivors in Uterine Cancer Empowered by Exercise and a Healthy Diet’ (SUCCEED), a behavioral lifestyle intervention at University Hospitals Case Medical Center (ClinicalTrials.gov Identifier: NCT00732173). This intervention aimed to improve nutritional and exercise behaviors over 16 group sessions in 6 months using the underpinnings of social cognitive theory. Five sessions were dedicated to improving EC patients’ diet quality including: 1) a session focusing on increasing intake of fruits and vegetables; 2) a session focusing on increasing whole grains and dairy; 3) a session on understanding food labels and choosing foods lower in fat and sodium and higher in fiber and calcium; 4) a session on modifying recipes to lower fat and sodium and how to plan meals in advance; and, 5) a session on strategies for choosing healthful foods while eating out and in social situations [27]. 11 out of the 15 EC patients (73.3%) enrolled in Wave 3 of SUCCEED consented to participate in the neuroimaging substudy. Under the main study randomization process, 8 of these patients were randomized to the intervention and 3 were randomized to the control group. The control group received usual care (UC), which consisted of an informational brochure (‘Healthy Eating and Physical Activity Across Your Lifespan’ booklet available at: http://www.win.niddk.nih.gov). Because there were only 3 EC patients randomized to the control group, we do not include the control group in these analyses. Patients had their height and weight measured at baseline and 6 months after the start of the intervention (i.e., after all 16 sessions were completed). Written informed consent was obtained from all study patients prior to their participation in the research study. Through this process, participants provided consent to publish findings from the fMRI data (e.g., brain images). The protocol was approved by the Institutional Review Board of University Hospitals Case Medical Center and the Case Comprehensive Cancer Center.

### fMRI procedures

Patients were instructed to fast overnight and underwent structural and functional MRI scans from approximately noon to 1:30 PM (pre-meal, fasted scan). Then, patients were provided a 750 kcal standardized meal prepared by the Dahms Clinical Research Unit (DCRU) Metabolic Kitchen at University Hospitals consisting of a sandwich (turkey, roastbeef or vegetarian), a serving of fruit, a serving of vegetables, yogurt or cottage cheese and a beverage (caffeine-free diet soda or water). Menu choices were balanced for macronutrient content. Patients were instructed to eat to satiation and, any remaining food was returned to the DCRU to be weighed and to provide an estimate the amount of energy (kcals) consumed. Approximately 25 to 30 minutes after the first scan, patients underwent the second set of functional scans (post-meal, fed scan). Because this study was part of a larger trial (SUCCEED), counterbalancing pre- and post-meal state by performing these scans on separate dates was not feasible.

Prior to scanning, a food preference assessment was administered whereby participants rated photograph flash cards of 74 foods (PCI Education, San Antonio, TX) that included fruits, vegetables, desserts, meats, snacks, breads and pastas on a 5-point Likert scale from ‘dislike’ (1) to ‘like’ (5). The food preference assessment photographs were different from the images used in the fMRI task. Food preference (‘liking’) ratings for high-calorie (e.g., cakes, cookies, potato chips, hot dogs) and low-calorie (e.g., fruits, vegetables) foods were compared between groups. Immediately before each scan, participants were asked to answer the question, “How hungry are you right now?’, using a Likert Scale response ranging from “Starving” (1) to “So Full You Could Burst” (10).

### fMRI experimental task

Changes in blood oxygen level-dependent (BOLD) contrast were measured using a blocked-design, perceptual discrimination task whereby patients indicated whether side-by-side color images of high-calorie food (e.g., cake, doughnuts, chips, fries), low-calorie food (fresh fruits and vegetables) and non-food objects (e.g., furniture, cars) were the “same” or “different” using a button press. The side-by-side images were from the same category (i.e., high-calorie, low-calorie or non-food). Each image was presented only once. All images including the non-food objects were created and modified for consistent size, brightness, and resolution (additional details can be found in [48]). Images were presented in 2 runs per session (pre-meal and post-meal) and were comprised of 8 blocks (21 seconds each with a 14-second rest between blocks) with 6 image pairs per block. Each run presented blocks of high-calorie foods, low-calorie foods and non-food in a counterbalanced order. Stimulus duration was set at 2250 ms with a 1250 ms interstimulus interval. The same/different tasks were selected to ensure participants were attending to the stimuli. The average task accuracy was similar during the pre-meal (97.0% ± 0.01%) and post-meal (98.3% ± 0.01%) scans at baseline; and, during the pre-meal (96.5% ± 0.01%) and post-meal (97.9% ± 0.02%) scans post-treatment. This paradigm has previously been shown to activate the lateral OFC, insula, hypothalamus, thalamus and amygdala in response to food cues [48] and differentiate neural response to food cues between lean and obese individuals [42].

### fMRI data acquisition and analyses

Data was acquired on a Wide-Bore (Magnetom) Verio 3.0 T MRI scanner (Siemens Medical Solutions, Malvern, PA) with a bore width of 70 cm and 550 lb weight capacity equipped with a 12-channel receiver head coil, an audio/visual system (Avotec, Inc., Stuart, FL) and an integrated four button response device (Lumina) at University Hospitals Case Medical Center and the Case Center for Imaging Research. Stimulus presentation was controlled by a computer synchronized to the 3.0 T operation using EPRIME (Psychology Software Tools, Inc.; http://www.pstnet.com/eprime). Functional images were acquired using a gradient- echo single-shot echo-planar sequence over 36 contiguous axial sequence slices aligned parallel to AC-PC plane with an inplane resolution of 3.4 × 3.4 × 3 mm (TR = 1950, TE = 22 ms, flip angle = 90°). BOLD activation data was acquired during two runs (5:01 minutes, 157 EPI) per session. 2D T1-weighted radio frequency spoiled gradient echo images (TR = 300, TE = 2.47 ms, FOV = 256, matrix = 256 × 256, flip angle = 60°, NEX = 2) in the same locations as the echo-planar data for in-plane registration and high resolution 3D structural images (3D MPRAGE, contiguous, sagittal acquisition, 176 images with 1 mm isotropic voxels, TR = 2500, TE = 3.52 ms, TI = 1100, FOV = 256, matrix = 256 × 256, flip angle = 12°, NEX = 1) for Talairach normalization and anatomical overlay were collected during the pre-meal session.

Image processing, statistical analyses and tests of statistical significance were performed using Brainvoyager QX v2.3.1 (Brain Innovation, Maastricht, The Netherlands). Preprocessing steps included trilinear 3D motion correction, 2D spatial smoothing with a Gaussian filter with full width half-maximum (FWHM) of 7 mm and high-pass filter temporal smoothing/linear trend removal. High-resolution functional 2D images were aligned to 3D anatomical images for display and localization using piecewise linear transformation into a proportional 3D grid defined by Talairach and Tournoux [49] and were coregistered with the high-resolution 3D data set and resampled to 3 mm^3^ voxels. Motion correction parameters were added to the design matrix and any movement >2 mm along any x-, y-, or z-axis was discarded (<1% was discarded).

Normalized data sets were entered into a random effects general linear model (GLM) analysis for the pre-meal and post-meal scans to compare the following contrasts: high-calorie vs. non-food; low-calorie vs. non-food and high-calorie vs. low-calorie foods. Resulting statistical maps were corrected for multiple comparisons using whole brain cluster-based threshold correction [50,51]. This cluster-correction approach allows for correction of multiple comparisons to reduce Type I errors while enabling the detection of true activations by exploiting the theory that areas of activation tend to stimulate changes over spatially contiguous groups of voxels (vs. over sparsely isolated voxels). More specifically, the cluster correction was performed using the “ClusterThresh” Plugin with 1,000 MonteCarlo simulations for each contrast map in BrainVoyager QX v2.3.1. We used an initial (uncorrected) threshold of p < 0.005 and a minimum contiguous cluster correction applied to each contrast map ranging from 6 to 15 voxels (162–405 mm^3^) to provide a family-wise error correction at p < 0.05.

In addition, we evaluated interactions of session (baseline vs. post-treatment) by condition contrast (high-calorie vs. nonfood; low-calorie vs. nonfood; high-calorie vs. low-calorie) for pre-meal and post-meal scan data. To visualize the interaction effects, we conducted secondary analyses whereby the magnitude of the BOLD effect (i.e., the mean beta value) was extracted for each participant for statistically significant regions (as identified in the primary analyses described above). Then, one-way ANOVAs were performed using the Statistical Package for Social Sciences (SPSS) v17.0 (Chicago, IL) to examine differences between high-calorie vs. non-food and low-calorie vs. non-food contrasts for each region by session type (baseline or post-treatment). We also explored potential correlations between mean changes in brain activation (i.e., the mean beta value) in significant regions from the high-calorie vs. non-food contrast and percent weight change at PostTx (i.e., 6 months after the SUCCEED intervention started). In addition, we evaluated potential correlations between mean changes in activation in these regions and high-calorie food preference (‘liking’) ratings.

## Results

### Demographic and behavioral data

Characteristics of the SUCCEED Neuroimaging Substudy population are shown in Table 1. The majority of the EC patients in the treatment (Tx) group were Stage I, Caucasian and, on average, were approximately 55 years old. At baseline, weight in the Tx group was, on average, approximately 205 lbs (204.5 ± 55.8 lbs), which would place most patients in Class II Obesity (BMI ≥ 35.0 kg/m^2^) per the World Health Organization criteria [52]. After 6 months in the SUCCEED intervention, the Tx group lost, on average, approximately 5 lbs (4.66 ± 6.65 lbs) or over 3 percent of their initial body weight (-3.4% ± 2.7%).

**Table 1:** Baseline Characteristics of the SUCCEED Neuroimaging Substudy Population

	**Treatment Group (Tx)**
	**(n = 8)**
Age (years)	54.5 (7.4)^2^
Caucasian	7 (87.5%)
Married or Living with Partner	7 (87.5%)
Stage I Endometrial Cancer	7 (87.5%)
Weight (lbs)	204.5 (55.8)
BMI (kg/m^2^)	35.8 (8.1)
Pre-Meal Hunger^1^	3.3 (1.7)
Post-Meal Hunger^1^ (‘Satiety’)	6.6 (1.1)
Fast Time (hrs since last ate)	10.1 (6.6)
Meal Energy (kcal) Consumed	511.2 (59.9)
High-Calorie Food Preference	3.84 (0.47)
Low-Calorie Food Preference	4.11 (0.33)

^1^Hunger was assessed based upon patient response to the following question: ‘How hungry are you right now?’ using a 10 point Likert response scale ranging from ‘Starving’ (1) to ‘So Full You Could Burst’ (10).

^2^Values in ( ) represent the s.d. of the mean or percentages, as applicable.

Hunger rankings post-meal were significantly higher than pre-meal at baseline (Table 1: post-meal: 6.6 ± 1.1 vs. pre-meal: 3.3 ± 1.7; p = 0.0003). Post-intervention (PostTx) post-meal hunger rankings were also higher than pre-meal hunger rankings (pre-meal hunger: 2.9 ± 1.6; post-meal hunger: 6.6 ± 0.9; p = 0.0002). Food preference (‘liking’) ratings for high-calorie (3.84 ± 0.47) and low-calorie (4.11 ± 0.33) foods were similarly high and were not significantly different (p = 0.20). The fasting time exceeded 8 hours, on average, and although the length of the fast was greater at PostTx (13.7 ± 5.3 hrs) compared to baseline (10.1 ± 6.6 hrs), the difference was not statistically significant (p = 0.24). The amount of energy consumed from the standardized meal was also similar between groups at baseline (Table 1) and post-intervention (Tx: 552.8 ± 168.7 kcals; UC: 484.9 ± 93.4 kcals).

### Baseline fMRI results

Significant functional activations found in the treatment (Tx) group at baseline (pre-treatment/intervention) for high-calorie vs. non-food, low-calorie vs. non-food and high- vs. low-calorie food contrasts are provided in Table 2. In the pre-meal condition (fasted state), we found significant increased activation when comparing high-calorie vs. non-food cues in several regions including the dorsolateral prefrontal cortex (DLPFC: BA = 9; -18, 49, 31), OFC (BA = 47; Bilateral: -47, 32,-7; 29,32,-7) and medial frontal gyrus (BA = 6: -9,28,37; BA = 10: -8,56,19). Increased activation was also seen with low-calorie vs. non-food contrasts in the fasted state in several regions including the claustrum (24,12,15), putamen (-24,0,16) and caudate (bilateral: -8,7,14; 13,-2,17). Interestingly, when comparing high- vs. low-calorie food cues, we observed a significant decreased activation in the anterior cingulate (21,39,15) in the fasting state.

**Table 2:** **Functional Activations for High-Calorie, Low-Calorie and Non-Food Object Contrasts at*Baseline*in Obese Endometrial Cancer Patients Enrolled in a 16-Session Group Behavioral Lifestyle Intervention**

	** Brain Region (Hemisphere)**	**Pre-Meal (Fasted)**					**Post-Meal (Fed/Satiated)**				
		**Peak Voxel**					**Peak Voxel**				
		**x**	**y**	**z**	Cluster Size*	***t***	**x**	**y**	**z**	Cluster Size*	***t***
**↑ Activation**	*High Calorie vs. Low Calorie Food*										
	Insula (BA = 13)						44	-11	18	524	5.67
	Cingulate Gyrus (BA = 31)						22	-21	44	491	6.04
	Precentral Gyrus (BA = 4)						53	-4	15	422	5.49
	*High Calorie vs. Non-Food Object*										
	OFC (Bilateral; BA = 47)	-47	32	-7	839	14.5					
	Medial Frontal Gyrus (BA = 6)	-9	28	37	897	11.6					
	Anterior PFC (BA = 10)	-8	56	19	825	10.4					
	Dorsolateral PFC (BA = 9)	-42	3	28	994	10.4					
	Dorsolateral PFC (BA = 46)	45	32	11	937	7.93					
	Middle Frontal Gyrus (BA = 8)	-39	26	42	648	8.27					
	Thalamus (Bilateral)						9	-16	3	847	5.00
	Posterior Cingulate (BA = 29)						-7	-45	18	491	5.24
	Precuneus (BA = 7)						-24	-77	47	535	7.65
	*Low Calorie vs. Non-Food Object*										
	Putamen	-24	0	16	756	9.35					
	Claustrum	24	12	15	830	9.89					
	Caudate	-8	7	14	783	7.64					
	Caudate	13	-2	17	296	5.17					
	Anterior PFC (BA = 10)	-32	64	4	635	8.70					
	Superior Frontal Gyrus (BA = 8)	-22	19	46	517	8.03					
**↓ Activation**	*High Calorie vs. Low Calorie Food*										
	Anterior Cingulate	21	39	15	766	-10.9					
	*High Calorie vs. Non-Food Object*										
	No regions survived threshold										
	*Low Calorie vs. Non-Food Object*										
	Culmen						22	-47	-12	639	-6.72

x, y, z = coordinates in Talairach space; t statistic for peak voxel; *Cluster size is reported in mm^3^. Results are whole brain cluster corrected for multiple comparisons; threshold α < 0.05 (min. cluster size: 10-36 voxels).

At baseline, when comparing high-calorie vs. non-food images in the fed state, we found significant increased activation in the thalamus (bilateral: 9,-16,3; -4,-14,8), posterior cingulate (BA = 29:-7,-45,18) and precuneus (BA = 7:-24,-77,47). When comparing high- vs. low-calorie food cues, we observed significant increased activation in the insula (BA = 1344,-11,18), cingulate gyrus (BA = 31: 22,-21,44) and precentral gyrus (53,-4,15). In the low-calorie vs. non-food contrast in the fed state, we found a significant decreased activation in the culmen (22,-47,-12).

### Post-Treatment (PostTx) fMRI results

Significant functional activations found at the post-intervention (PostTx) scan for high-calorie vs. non-food, low-calorie vs. non-food and high- vs. low-calorie food contrasts are provided in Table 3. In the fasted state, when comparing high-calorie vs. non-food images, we observed increased activation in the culmen (BA = 10; -4,-57,2) and middle frontal gyrus (BA = 6; -53,7,44) and, when comparing high- to low-calorie food images, we found increased activation in the declive (-48,-83,-20; 49,-64,-9). In the post-meal state, when comparing high- vs. low-calorie images, we found increased activation in the insula (BA = 13; 42, 11, 0), anterior cingulate (BA = 32; -16,44,-4), inferior frontal gyrus (BA = 46; 44,38,12) and middle frontal gyrus (BA = 9; BA = 10) and, when comparing high-calorie to non-food images, we observed decreased activation in the caudate (tail;18,-37,20) and claustrum (29,-20,16). When comparing low-calorie vs. non-food images post-meal, decreased activation was observed in the insula (BA = 13;-34,-15,31), cingulate gyrus (BA = 31; -8,-23,43), anterior cingulate (BA = 24; -9, 32, 13) and middle frontal gyrus (BA = 10; -7,55,12).

**Table 3:** **Functional Activations for High-Calorie, Low-Calorie and Non-Food Object Contrasts at*Post-Treatment*in Obese Endometrial Cancer Patients Enrolled in a 16-Session Group Behavioral Lifestyle Intervention**

	** Brain Region (Hemisphere)**	**Pre-Meal (Fasted)**					**Post-Meal (Fed/Satiated)**				
		**Peak Voxel**					**cPeak Voxel**				
		**x**	**y**	**z**	Cluster Size*	***t***	**x**	**y**	**z**	Cluster Size*	***t***
**↑ Activation**	*High Calorie vs. Low Calorie Food*										
	Declive	-48	-83	-20	457	8.70					
	Declive	-19	-64	-9	233	4.87					
	Inferior Frontal Gyrus (BA = 46)						44	38	12	907	8.97
	Middle Frontal Gyrus (BA = 9)						41	18	31	575	9.75
	Middle Frontal Gyrus (BA = 10)						40	62	8	479	12.5
	Anterior Cingulate (BA = 32)						-16	44	-4	403	7.63
	Insula (BA = 13)						42	11	0	182	5.94
	*High Calorie vs. Non-Food Object*										
	Culmen (BA = 10)	-4	-57	2	196	5.04					
	Middle Frontal Gyrus (BA = 6)	-53	7	44	171	4.60					
	*Low Calorie vs. Non-Food Object*										
	No regions survived threshold										
**↓ Activation**	*High Calorie vs. Low Calorie Food*										
	Medial Frontal Gyrus (BA = 10)	5	55	12	292	-9.68					
	*High Calorie vs. Non-Food Object*										
	Precentral Gyrus (BA = 4)	48	-6	42	379	-4.86					
	Caudate Tail						18	-37	20	735	-10.7
	Claustrum						29	-20	16	492	-7.36
	*Low Calorie vs. Non-Food Object*										
	Culmen	9	-58	-8	457	-7.76					
	Declive	22	-60	-13	680	-8.40					
	Cingulate Gyrus (BA = 31)						-8	-23	43	672	-11.6
	Postcentral Gyrus (BA = 3)						-56	-10	22	701	-15.3
	Insula (BA = 13)						-34	-15	21	839	-11.3
	Anterior Cingulate (BA = 24)						-9	32	13	516	-8.84
	Medial Frontal Gyrus (BA = 10)						-7	55	12	640	-8.46

x, y, z = coordinates in Talairach space; t statistic for peak voxel; *Cluster size is reported in mm^3^. Results are whole brain cluster corrected for multiple comparisons; threshold α < 0.05 (min. cluster size: 6-38 voxels).

### Changes in fMRI activation post-treatment compared to baseline

Significant functional activations found when comparing post-intervention (PostTx) versus Baseline (PreTx) for high-calorie vs. non-food, low-calorie vs. non-food and high- vs. low-calorie food contrasts are provided in Table 4. In the pre-meal condition, we found that the Tx group showed significant decreased activation PostTx compared to PreTx in only one region, the superior frontal gyrus (BA = 6; 38,39,30), for the high-calorie vs. non-food contrast. No other regions survived the significance threshold for all other (low calorie vs. non-food, high-calorie vs. low-calorie) pre-meal contrasts examined.

**Table 4:** **Functional Activations for High-Calorie, Low-Calorie and Non-Food Object Contrasts*Post-Treatment vs. Baseline (Pre-Treatment)*in Obese Endometrial Cancer Patients in a 16-Session Behavioral Lifestyle Intervention**

	** Brain Region (Hemisphere)**	**Pre-Meal (Fasted)**					**Post-Meal (Fed/Satiated)**				
		**Peak Voxel**					**Peak Voxel**				
		**x**	**y**	**z**	Cluster Size*	***t***	**x**	**y**	**z**	Cluster Size*	***t***
**POST < PRE TREATMENT**	*High Calorie vs. Low Calorie Food*										
	No regions survived threshold										
	*High Calorie vs. Non-Food Object*										
	Superior Frontal Gyrus (BA = 6)	38	39	30	416	-4.72					
	Lateral Globus Pallidus						20	-13	6	972	-9.45
	Cingulate Gyrus (BA = 31)						-9	-23	34	968	-6.63
	Thalamus						-14	-24	5	738	-5.63
	Posterior Cingulate (BA = 29)						-8	-45	16	583	-6.52
	Precuneus (BA = 31)						-16	-44	30	811	-5.85
	Claustrum						37	-6	6	911	-6.62
	*Low Calorie vs. Non-Food Object*										
	Insula (Bilateral; L; BA = 13)						38	-22	11	613	-5.44
	Insula (Bilateral; R; BA = 13)						-40	-13	14	691	-6.64
	Precentral Gyrus (BA = 4)						-52	-12	24	963	-10.6
	Middle Temporal Gyrus (BA = 39)						-39	-50	6	568	-4.73
**POST > PRE TREATMENT**	*High Calorie vs. Low Calorie Food*										
	Superior Temporal Gyrus (BA = 22)						-48	11	-5	758	5.72
	Superior Frontal Gyrus (BA = 10)						21	63	27	607	5.90
	*High Calorie vs. Non-Food Object*										
	No regions survived threshold										
	*Low Calorie vs. Non-Food Object*										
	No regions survived threshold										

x, y, z = coordinates in Talairach space; t statistic for peak voxel; *Cluster size is reported in mm^3^. Results are whole brain cluster corrected for multiple comparisons; threshold α < 0.05 (min cluster size: 15–36 voxels).

**Figure 1:**
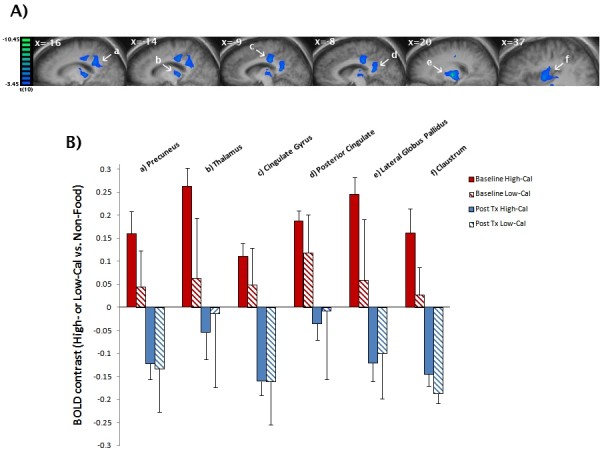
**Post-Treatment Compared to Baseline for High-Calorie vs. Non-Food Post-Meal Contrast in Obese Endometrial Cancer Patients Receiving a 16 Session/6-month Behavioral Lifestyle Intervention. A**) Main effect (saggital view) of significant (whole brain cluster corrected, p < 0.05) decreased activations (blue) post-treatment (PostTx) compared to baseline (PreTx) for high-calorie vs. non-food objects in the fed state: **a**) precuneus; **b**) thalamus; **c**) cingulate gyrus; **d**) posterior cingulate; **e**) lateral globus pallidus; **f**) claustrum. **B**) Mean BOLD effect (mean beta value) at PostTx (blue) and PreTx (red) by calorie condition. In these regions, high-calorie vs. non-food contrasts were significantly (p < 0.001) lower PostTx vs. PreTx (solid blue vs. solid red) (see text).

**Figure 2:**
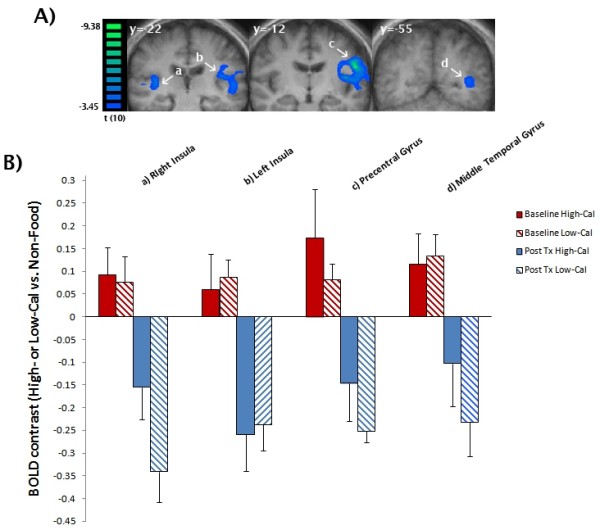
**Post-Treatment Compared to Baseline for Low-Calorie vs. Non-Food Post-Meal Contrast in Obese Endometrial Cancer Patients Receiving a 16 Session/6-month Behavioral Lifestyle Intervention. A**) Main effect (coronal view) of significant (whole brain cluster corrected, p < 0.05) decreased activation (blue) observed post-treatment (PostTx) compared to baseline (PreTx) for high-calorie vs. non-food objects in the fed state: **a**) insula (bilateral); **b**) postcentral gyrus; and, **c**) middle temporal gyrus. **B**) Mean BOLD effect (mean beta value) at PostTx (blue) and PreTx (red) by calorie condition. In these regions, low-calorie vs. non-food contrasts were significantly (p < 0.001) lower PostTx vs. PreTx (hatched blue vs. hatched red) (see text for details).

In the post-meal condition, we found several regions which showed significant decreased activation after the intervention (PostTx) compared to baseline (PreTx) (Table 4). Specifically, we found a significant reduction in activation in the high-calorie vs. non-food contrast for the lateral globus pallidus (20,-13,6), cingulate gyrus (BA = 31; -9,-23,34), thalamus (-14,-24,5), posterior cingulate (BA = 29; -8,-45,16), precuneus (BA = 31; -16,-44, 30) and claustrum (37,-6,6) (Figure 1, panel A). For the low-calorie vs. non-food contrast, we found significant decreased activation PostTx compared to PreTx in the insula (bilateral BA = 13; 38,-22,11; -40,-13,14), precentral gyrus (BA = 4; -52,-12,24) and middle temporal gyrus (BA = 39; -39,-50,6) (Figure 2, panel A). In the high- vs. low-calorie contrast, we observed a significant increased activation PostTx compared to PreTx in the anterior (BA = 22; -48,11,-5) and superior frontal gyrus (BA = 10; 21,63,27).

### Exploratory analyses in statistically significant regions

Post-hoc analyses were performed to confirm the initial findings (discussed above) and illuminate interaction effects. Figure 1 (panel B) provides the mean BOLD effect (mean beta value) for regions found to be significant in the PostTx (blue) vs. PreTx (red) high-calorie vs. non-food contrast. As illustrated in Figure 1 (panel B), the high-calorie vs. non-food contrasts were significantly lower Post-Tx vs. PreTx (solid blue vs. solid red) for the precuneus (F = 22.40, p < 0.001), posterior cingulate (F = 28.23, p < 0.001), thalamus (F = 20.12, p = 0.001), cingulate gyrus (F = 41.58, p < 0.001), lateral globus pallidus (F = 44.89, p < 0.001) and claustrum (F = 27.54, p < 0.001). As illustrated in Figure 2 (panel B), the low-calorie vs. non-food contrasts were significantly lower in Post-Tx vs. PreTx (solid blue vs. solid red) for the insula (38,-22,11: F = 22.91, p < 0.001; -40,-13,14: F = 22.40, p < 0.001), postcentral gyrus (F = 59.26, p < 0.001) and middle temporal gyrus (F = 16.91, p < 0.001).

**Figure 3:**
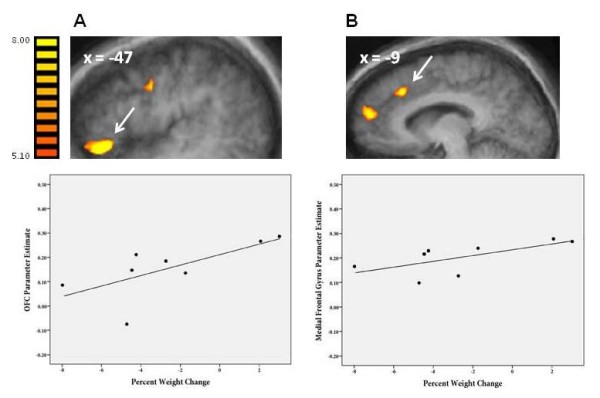
**Correlations Between Percent Weight Change and Activations from the High-Calorie vs. Non-Food Contrast at Baseline (Pre-meal) in Obese Endometrial Cancer Patients Receiving a 16 Session/6-month Behavioral Lifestyle Intervention. A**) Top Row: Increased activation in orbitofrontal cortex (OFC) (saggital view; parameter estimate is mean beta value); Bottom Row: Correlation between OFC activation and percent weight change (r = 0.69, p < 0.05): **B**) Top Row: Increased activation in medial frontal gyrus (MFG); Bottom Row: Correlation between MFG activation and percent weight change (r = 0.66, p < 0.05).

In addition, we also explored potential correlations between mean changes in brain activation from significant regions from the high-calorie vs. non-food contrast and percent weight change (Figure 3). We found positive correlations between percent weight change and differential activation at baseline in frontal regions including the OFC (p = 0.69; p < 0.05; Figure 3A) and medial frontal gyrus (r = 0.66; p < 0.05; Figure 3B), indicating that increased activation in these frontal regions, at baseline, was associated with less successful weight loss. Positive correlations between high-calorie food preference (‘liking’) ratings and increased activation in these two regions were also found (OFC: r = 0.62; p < 0.05; medial frontal gyrus: r = 0.54, p < 0.05). When examining PostTx separately, none of the regions listed in Table 3 for the high-calorie vs. non-food contrast were significantly correlated with percent weight change. When comparing PostTx to baseline, none of the significant regions listed in Table 4 for the high-calorie vs. non-food contrast were significantly correlated with percent weight change.

## Discussion

We found that the neural response to high-calorie visual food cues in obese endometrial cancer (EC) survivors, at baseline, was similar to that previously reported for the general adult obese population in both fasted (pre-meal) and fed (post-meal) states. In addition, we found that obese EC patients had decreased activation to high-calorie food cues after a 6 month behavioral lifestyle intervention compared to baseline in regions associated with food reward and motivation.

Our results in obese EC patients at baseline are consistent with previous findings in the general obese population, which are nicely summarized in a recent review by Carnell et al. [35]. More specifically, previous fMRI studies have shown that obese compared to normal weight individuals have greater activation in brain regions involved in food motivation and reward processing including the insula, lateral orbitofrontal cortex (OFC), amygdala, dorsal striatum and putamen in response to visual high-calorie/palatable food stimuli even after eating a meal [38,39,41,42]. In our study, at baseline, we found that obese EC patients, who appear to be slightly more obese than the general adult female population in the U.S. [13-15], had significant increased activation even after consuming a meal in the insula (BA = 13), cingulate gyrus (BA = 31) and precentral gyrus (BA = 4) for the high- vs. low-calorie contrast and, increased activation in the thalamus, posterior cingulate (BA = 29) and precuneus (BA = 7) for the high-calorie vs. non-food contrast. Because this was the first study in obese EC survivors, we also explored the low-calorie vs. non-food contrast and found, at baseline, increased activation in the putamen, claustrum, caudate, anterior PFC (BA = 10) and SFG (BA = 8) in the fasted state but no significant increased activations in the fed state. The increased activation we observed in the fasted state with low-calorie food cues was likely a function of the patients’ heightened hunger (due to fasting greater than 8 hours, on average). We hypothesize that, in the fed state, our lack of increased activation with low-calorie food cues in obese EC patients mimics findings in the general obese population in that exaggerated food-cue reactivity is more pronounced with high-calorie food cues [41]. However, the differential activations we observed could be associated with alternative interpretations. Activations are based upon predicted or anticipatory responses from combined sensory data and prior experiences and, anticipatory and consummatory responses may be different and affected by mood [53]. Although the cingulate gyrus has been hypothesized to affect emotional self-control, particularly as it relates to dietary restraint [54], activation could also be a function of anticipatory preferences as many pleasant stimuli activate this region [55]. Moreover, although increased activation in the dlPFC has been correlated with eating restraint, the dlPFC may spontaneously engage self-control mechanisms when individuals view food pictures to arrive at perceived socially acceptable (or ‘appropriate’) responses [56,57].

To our knowledge, there is only one prior *prospective* neuroimaging study that has evaluated response to visual food cues in obese patients (and normal weight controls) before and after a behavioral lifestyle intervention [47]. In this study, they found significant decreased activation post-treatment (Session 2: after the 12-week ‘EatRight Lifestyle Weight Management Program’, which has the overarching theme to encourage replacement of high-energy-dense foods by low-energy-dense foods through weekly educational sessions) compared to baseline (Session 1: within 3 weeks of starting the EatRight Program) in the obese patients’ response to high-calorie vs. non-food cues in brain regions involved in reward and attentional processing including the medial prefrontal cortex (BA = 32), precuneus (BA = 7), and posterior cingulate (BA = 23, BA = 31). In our study, we found similar decreased activations with high-calorie vs. non-food contrasts in obese EC patients (treatment group) in the fed state when comparing post-treatment to baseline in the precuneus (BA = 31) and posterior cingulate (BA = 29) as well as the thalamus, lateral globus pallidus and cingulate gyrus (BA = 31), which are regions shown to be involved in reward, motivation, emotional processing and feeding decisions [58,59]. We also found decreased activation for the low-calorie vs. non-food contrast in the fed state in the insula (bilateral, BA = 13), which is involved in taste processing and, the precentral gyrus (BA = 4), which is involved in pre-motor cortex planning and execution. Although our obese EC patient population was slightly heavier at baseline (mean BMI: 35.8 ± 8.1 kg/m^2^) than that of the adult obese population of Murdaugh et al. [47] (mean BMI: 32.9 ± 3.8 kg/m^2^), we observed a similar decrease in percent weight loss (3.4% ± 2.7%) as that found in Murdaugh et al. [47] (3.5% ± 2.4%). Therefore, it appears that both behavioral lifestyle interventions, which aimed to increase the intake of low-calorie/nutrient-rich foods (e.g., fruits and vegetables) while concomitantly decreasing the intake of high-calorie/nutrient-weak foods (e.g., chips and sweets) produced similar results in terms of weight loss and decreased neural activation in food motivation and reward regions in response to high-calorie food cues after the intervention compared to pre-treatment.

A few studies have prospectively evaluated response to visual food cues before and after bariatric and gastric banding surgery. Although surgical and behavioral therapy studies are not directly comparable due, in part, to the substantially higher weight loss in surgical therapies (ranging from 11.8% [60] to 25.2% ± 8.4% [61]), we observed differential activation in some of the same brain regions in our study with only 3.4% ± 2.7% weight loss. For example, Ochner et al. [60], found decreased activation in the precuneus, posterior cingulate and thalamus in response to high-calorie food cues in the fed state among Class III obese patients (n = 10; mean BMI: 45.0 ± 5.0 kg/m^2^) 1-month after bariatric surgery compared to 1-month prior to their surgery. Furthermore, Bruce at al. [61] found that 12 weeks after gastric banding surgery, Class III obese patients (n = 10; mean BMI: 40.6 ± 1.96 kg/m^2^) showed decreased activation in food motivation and reward regions including the insula, inferior middle gyrus and middle frontal gyrus. Interesting, we and Bruce et al. [61] found increased activation post-treatment compared to baseline in the superior frontal gyrus (SFG), which could suggest increased self-regulation post-treatment but might also represent the continued struggle with cognitive control when viewing high-calorie food cues. Bruce et al. [61] also observed increased activation in the middle frontal gyrus which is a region previously found to have leptin-reversible increased neural activity in response to food cues following weight loss [54]. Rosenbaum et al. [54] suggested that weight loss reflects a state of leptin-deficiency and a phenotype with greater emotional and sensory responsiveness to food cues, make maintaining weight loss more difficult. The results with leptin administration observed by Rosenbaum et al. [54] seem to conflict with the results we and others have observed in behavioral and surgical weight loss interventions. Nevertheless, we believe our results, taken together with the results of other studies that have prospectively examined response to visual food cues before and after behavioral lifestyle [47] and surgical interventions [60,61], suggest these types of interventions may help to dampen the response to high-calorie food cues, although more aggressive and/or longer-term interventions may be required to fully alleviate the incentive salience of high-calorie food cues corresponding to the increased activation found post-treatment relative to pre-treatment in attentional processing areas.

When evaluating the post-treatment (PostTx) scan separately, we found increased activation in only a few regions including the declive, culmen and middle frontal gyrus in the fasted state when comparing high-calorie vs. non-food images. In the post-meal state, when comparing high- vs. low-calorie images, we found increased activation predominantly in the frontal regions including the middle frontal gyrus and inferior frontal gyrus. Furthermore, we found decreased activation post-meal in several brain regions associated with food reward and motivation (insula, anterior cingulate, cingulated gyrus, middle frontal gyrus, inferior frontal gyrus) when comparing low-calorie vs. non-food images. Taken together, these results suggests that obese EC patients may be viewing food cues differently post-intervention with increased cognitive attention and, perhaps, less rewarding value, to high-calorie food images, particularly after satiation. We cannot compare our PostTx scan results to previous prospective lifestyle [47] and surgical [60,61] intervention studies because previous studies only present their post-treatment results relative to the pre-treatment condition, as the neural adaptations in response to the intervention may be better elucidated when comparing the post-treatment to the pre-treatment condition.

With regard to our exploratory studies evaluating correlations with weight loss, we found positive correlations between baseline activations in high-calorie vs. non-food contrasts and percent weight change similar to Murdaugh et al. [47]. However, our findings were limited to two frontal regions (OFC; medial frontal gyrus) while Murdaugh et al. [47] reported significant correlations in visual areas including the superior parietal lobe and middle frontal gyrus (BA = 8). When comparing PostTx to baseline scans, we did not observe any significant correlations between regions showing significant activation with high-calorie compared to non-food contrasts and percent weight loss. Murdaugh et al. [47] also did not report any significant correlations between percent weight change and activation in regions of interest at 12 weeks post-treatment (Session 2) compared to pre-treatment (Session 1) but they did observe significant negative correlations between percent weight change at 9 months (6 months after the intervention was completed) and change in activation (Session 1 minus Session 2) when comparing high-calorie vs. non-food images in several regions including the insula and thalamus. Bruce et al. [61] reported that they found no significant correlations between percent weight change and activation in regions of interest when examining visual food cues in obese adults before and after gastric banding surgery. Given the small sample size in our study and in Bruce et al. [61], our studies may have been underpowered to detect the significant correlations observed in Murdaugh et al. [47], which had over two times the number of patients. On the other hand, given the number of exploratory tests we performed without correcting for multiple testing, it is possible that the significant correlations we found at baseline were due to chance. Clearly, additional, larger prospective lifestyle and surgical intervention studies are needed to better elucidate the brain regions that may be most amenable to neural adaptations that drive sustained weight loss.

Overall, our results suggest the SUCCEED behavioral lifestyle intervention, which focused on replacing high-calorie/nutrient-weak foods (e.g., chips and sweets) with low-calorie/nutrient-dense foods (e.g., fruits and vegetables), may help reduce the rewarding value of high-calorie food cues, particularly after eating meal, in obese endometrial cancer survivors. However, given the increased activation we observed in frontal regions post-treatment compared to baseline, which could suggest increased self-regulation post-treatment but might also represent the continued struggle with cognitive control when viewing high-calorie food cues, more aggressive and/or longer-term interventions may be required to fully alleviate the incentive salience of high-calorie food cues. If the necessary neural adaptations in brain regions implicated in food reward and motivation could be maintained and long-term weight loss sustained, we could ultimately increase the survival of obese EC patients, who are at a very high risk of death from the co-morbidities associated with their obesity [14,17].

Major limitations of our study in obese EC patients include the lack of a control group and a very small sample size. Therefore, our results should be considered preliminary and interpreted with caution until replicated in a larger study. Nevertheless, the differential activations we observed at baseline and for post-treatment compared to baseline are consistent with other previously published studies conducted in the general obese population. Although we provided the obese EC patients with a 750 kcal standardized meal, they only consumed, on average, about 500-550 kcals. Patients had the ability to select the type of sandwich (turkey, roastbeef, vegetarian) but they did not have the opportunity to select the type of fruit and vegetable provided; and, the items most likely to remain uneaten were vegetables and condiments (mayonnaise, mustard). Nevertheless, patients reported that they were no longer hungry after the meal and their food preference ratings were high for both high- and low-calorie foods. Interestingly, previous studies in obese populations have provided much smaller meals (e.g., 250 kcal liquid meal [60]) and have found similar activations in response to high-calorie food cues, suggesting that the total calories consumed may not have a material effect.

## Conclusions

Our preliminary results suggest behavioral lifestyle interventions may help reduce high-calorie food reward in obese endometrial cancer survivors. Although confirmation of our findings await additional studies, our results are consistent with those recently published by Murdaugh et al. [47] in the general obese population and, taken together, suggests lifestyle interventions may effectively target brain regions involved in reward processing and attentional control.

## Competing interests

The authors declare that they have no competing interests.

## Author contributions

N.L.N. conceived of the neuroimaging study, developed the overall research plan, performed study oversight, statistical analysis and wrote the paper. A.D. provided oversight of all neuroimaging aspects including research design, statistical analysis and inference and contributed to the writing of this manuscript. J.T. provided oversight of the fMRI design and contributed to writing of this manuscript. H.F. helped conduct the SUCCEED study intervention, data collection and contributed to writing of the manuscript. V.V. conceived, developed and oversaw the SUCCEED study intervention and contributed to the writing of this manuscript. All authors read and approved the final manuscript.
